# Characterization of the Crustacean Methyl Farnesoate Transcriptional Signaling Genes

**DOI:** 10.3390/ijms27031215

**Published:** 2026-01-26

**Authors:** Vanessa L. Bentley, Jorge L. Pérez-Moreno, David S. Durica, Donald L. Mykles

**Affiliations:** 1 Department of Biology, Colorado State University, Fort Collins, CO 80523, USA; jperezmoreno@umass.edu; 2 Department of Biology, University of Massachusetts, Amherst, MA 01003, USA; 3 School of Biological Sciences, University of Oklahoma, Norman, OK 73019, USA; ddurica@ou.edu; 4 Bodega Marine Laboratory, University of California, Davis, Bodega Bay, CA 94923, USA

**Keywords:** crustacea, methyl farnesoate, Y-organ, molting, Methoprene-tolerant, steroid receptor coactivator, Krüppel homolog 1, Ecdysone response gene 93, CREB-binding protein, C-terminal-binding protein, transcription factor, bHLH

## Abstract

Methyl farnesoate (MF) is a sesquiterpenoid hormone that controls a variety of physiological processes in crustaceans, including morphogenesis, development, reproduction, and molting. MF action is mediated by a transcriptional signaling cascade consisting of *Methoprene-tolerant* (*Met*), *Steroid receptor coactivator* (*Src*), *Krüppel homolog 1* (*Kr-h1*), and *Ecdysone response gene 93* (*E93*) transcription factors (TFs), and transcriptional co-regulators *CREB-binding protein* (*CBP*) and *C-terminal-binding protein* (*CtBP*). Phylogenetic and sequence analyses revealed that these genes were highly conserved across pancrustacean species. Met and Src were characterized as basic helix-loop-helix, Period (Per)-Aryl Hydrocarbon Nuclear Translocator (ARNT)-Single-minded (Sim) protein (bHLH-PAS) TFs; Kr-h1 was characterized as a C_2_H_2_ zinc finger TF with seven zinc finger motifs; E93 was characterized as a helix-turn-helix, pipsqueak (HTH_Psq) TF. CBP was identified by several zinc finger-binding regions with Transcription Adaptor Zinc Finger 1 and 2, Really Interesting New Gene, Plant homeodomain, and Z-type zinc finger domains; the Kinase-inducible Domain Interacting-transcription factor docking site; the Bromodomain-acetylated lysine recognition and binding site; the histone acetyltransferase domain; and a C-terminal CREB-binding region containing a nuclear receptor co-activator-binding domain. CtBP had a dehydrogenase domain with arginine-glutamate-histidine catalytic triad. 81 *Met* contigs, 45 *Src* contigs, 136 *Kr-h1* contigs, 66 *E93* contigs, 60 *CBP* contigs, and 172 *CtBP* contigs were identified across pancrustacean taxa, including decapod crustaceans. Bioinformatic identification and annotation of these TFs and co-regulators in brachyuran Y-organ (YO) transcriptomes suggests that MF signaling influences YO ecdysteroidogenesis; functional tests in the YO are needed to establish causality.

## 1. Introduction

Juvenile hormone (JH) and methyl farnesoate (MF) are sesquiterpenoid hormones that control various physiological processes, including development, growth, molting, and reproduction in insects and crustaceans, respectively (reviewed in [1,2,3,4,5,6,7,8]). JH/MF action is mediated by the MEKRE93 signaling cascade of transcription factors (TFs) consisting of Methoprene-tolerant (Met), Krüppel homolog 1 (Kr-h1), and Ecdysone inducible protein 93F (E93) as the central components (reviewed in [9,10,11]). Transcriptional co-regulators CREB-binding protein (CBP) and C-terminal-binding protein (CtBP) can alter the expression of JH biosynthesis and signaling genes in insects [3,12,13].

Met serves as the insect JH receptor and is a critical regulator of insect reproduction and development (reviewed in [14,15,16,17,18,19,20]). Met, along with Steroid receptor coactivator (Src), are transcriptional regulators within the basic helix-loop-helix (bHLH) protein superfamily [21]. Members of this superfamily are characterized by a ~60 amino acid DNA-binding domain, followed by two helices separated by a variable loop region that facilitates dimerization [22,23,24,25]. Met and Src are classified in the bHLH-Period, Aryl Hydrocarbon Receptor Nuclear Translocator, and Single-minded (PAS) family, which has a conserved N-terminal bHLH domain followed by two PAS domains (PAS-A and PAS-B) and a C-terminal transactivation domain [14,20,26,27]. The PAS-B domain binds JH and JH analogs, such as methoprene and fenoxycarb [14,27]. In *Drosophila melanogaster* Met, eight amino acid residues within the PAS-B domain are essential for JH III binding [17,28]. The C-terminus is a highly variable and disordered region that contributes to the functional properties of family members [29,30]. Met forms a heterodimer with Src or other bHLH-PAS TFs, such as Cycle (CYC), Germ cell expressed (Gce), Ftz-F1-interacting steroid receptor coactivator (FISC), or Taiman (Tai) [1,14,27,31,32]. Tai acts as the obligatory DNA-binding partner of Met in *D. melanogaster*; this receptor complex recognizes an E-box sequence in the promoters of JH target genes, which leads to transcriptional activation [14,33,34]. In Src, the bHLH-PAS domain is followed by a receptor-interacting domain containing a conserved LxxLL motif that mediates ligand-dependent interactions, and a C-terminal transcriptional activation domain [35]. The transcriptional activity of Src can be modulated by histone acetylation, methylation, and the recruitment of co-regulators, such as CBP/p300, to the C-terminus [35].

Krüppel homolog 1 (Kr-h1) is the key TF regulating insect metamorphosis and reproduction [15,36]. Kr-h1 belongs to the largest group of TFs in higher eukaryotes: the Cys_2_-His_2_ (C_2_H_2_) zinc finger (Zf) proteins [37]. The two cysteines and two histidines coordinate binding of a zinc ion, thereby stabilizing a finger-like loop that enables DNA-binding [38,39,40]. In insects, the DNA-binding domain contains eight C_2_H_2_ Zf repeats [36], whereas in decapod crustaceans the DNA-binding domain contains seven C_2_H_2_ Zf repeats [41,42]. Multiple Kr-h1 isoforms are generated from alternative promoters within the *Kr-h1* locus [36]. Kr-h1 can be phosphorylated by protein kinase Cα to recruit co-repressor C-terminal binding protein (CtBP) to inhibit E93 expression in insects [13].

E93 encodes a helix-turn-helix, pipsqueak (HTH_Psq) TF that recognizes the GAAG response element in gene promoters to regulate chromatin accessibility in response to temporal and spatial cues [43,44,45,46,47]. E93 orchestrates gonadotrophic cycles in female yellow fever mosquitoes (*Aedes aegypti*) and tissue remodeling during metamorphosis in other insects [48,49,50,51,52,53,54,55].

cAMP response element binding protein (CREB)-binding protein (CBP) and its p300 paralog function as transcriptional co-activators with over 400 interaction partners identified [56]. CBP has histone acetyltransferase (HAT) activity that is essential for chromatin remodeling for metazoan growth and development [56]. The Cysteine/Histidine-rich 1 (CH1) region contains the Transcriptional Adaptor Zinc finger 1 (TAZ1) domain and the Kinase-Inducible Interacting (KIX) domain [57]. The KIX domain of CBP is a TF docking site with three α- and two 3_10_- helices forming two binding surfaces that determine the magnitude of a transcriptional response [57]. The CBP catalytic core consists of (1) a Bromodomain (BROMO) with an acetylated lysine recognition and binding site; (2) the Cysteine/Histidine-rich 2 (CH2) domain, which contains the Really Interesting New Gene (RING) and Plant Homeodomain (PHD) subdomains; (3) the HAT domain; and (4) the Cysteine/Histidine-rich 3 (CH3) domain, which contains the ZZ zinc finger (ZZ) and Transcriptional Adaptor Zinc finger 2 (TAZ2) [58,59]. Interactions with the TAZ2 domain bring TFs into proximity with the HAT domain, thereby contributing to acetylation-dependent regulation of chromatin structure [60]. At the N-terminus, the CREB-binding region contains co-activator binding sites within the nuclear co-activator binding domain (NCBD), which interacts with partners, such as Src and thyroid and retinoid receptors [58]. The C-terminus contains a glutamine- and proline-rich region that contributes to transcriptional activation and protein-protein interactions [56].

CtBP represses transcription by recruiting histone deacetylases and other co-repressor complexes to target genes and plays essential roles in development, tumorigenesis, and cell fate decisions [61,62]. CtBP contains a D2 hydroxyacid dehydrogenase (D2-HDH) domain [63]. CtBP typically occurs as a dimer but can assemble into higher-order oligomers, with tetramers exhibiting greater transcriptional repression activity [63,64]. The D2-HDH domain contains a Rossmann fold with a canonical GxGxxG motif for NAD(H) binding and a diagnostic Arginine-Glutamate-Histidine (REH) catalytic triad [63]. The D2-HDH domain is flanked by N-terminal and C-terminal domains. The N-terminal domain (NTD) contributes to the binding cleft that recognizes TF partners carrying conserved motifs (e.g., PxDLS repressor motif) [63]. Across bilaterians, the NTD is highly conserved in length and contains multiple putative post-translational modification sites, including those for phosphorylation and SUMOylation [64,65,66]. By contrast, the C-terminal domain (CTD) is a proline- and glycine-rich structurally disordered region that is a characteristic of hydroxyacid dehydrogenases [66]. CtBP isoforms either retain or lack the CTD, with both forms functionally competent [64,65]. In *D. melanogaster*, these isoforms differentially influence wing development, suggesting the selective modulation of gene targets [65]. Vertebrates possess multiple CtBP paralogs due to gene duplication, while invertebrates generally have a single CtBP gene [64]. In insects, multiple isoforms are generated by alternative mRNA splicing and promoter usage [64].

The MF MEKRE93 signaling transcriptional cascade regulates reproduction, metamorphosis, and molting in crustaceans (reviewed in [7,67,68]). MF stimulation of vitellogenesis and ovary maturation is mediated by *Met* and *Kr-h1* in the gazami crab (*Portunus trituberculatus*), Chinese mitten crab (*Eriocheir sinensis*), green mud crab (*Scylla paramamosain*), and whiteleg shrimp (*Litopenaeus vannamei*) [41,42,69,70,71,72,73]. MF inhibition of metamorphosis is associated with co-expression of *Sp-Met*, *Sp-Kr-h1*, and *Sp-E93* in *S. paramamosain* larvae, as partial knockdown of *Sp-Met* by *Sp-Met*-RNAi lowered *Sp-Kr-h1* and *Sp-E93* mRNA levels [72]. MF acting through *Met*, *Kr-h1*, and *E93* can accelerate molting by stimulating the synthesis of molting hormones (ecdysteroids) in the Y-organ (YO) [74,75,76,77,78].

MEKRE93 signaling and co-regulator genes have been characterized in relatively few decapod crustaceans (reviewed in [7]). Here we characterized transcripts encoding pancrustacean *Met*, *Src*, *Kr-h1*, *E93*, *CtBP* and *CBP* that were obtained from various databases, including CrusTome, a multi-species and multi-tissue transcriptome database of 189 crustacean species [79], CrustyBase [80,81], Crustacean Transcription Factor (CrusTF) [82], Crustacean Annotated Transcriptome (CAT) [83], and GenBank. Phylogenetic analysis and multiple sequence alignments (MSAs) determined evolutionary relationships and identified functional domains and motifs that can be used for accurate annotation. A similar strategy was used to characterize pancrustacean neuropeptide receptors, growth factor receptors, and AKG protein kinases [84,85,86,87]. Decapod sequences were emphasized, as this group includes species of ecological and economic importance [88,89,90]. The results constitute a comprehensive catalog of the MEKRE93 signaling and co-regulator genes for the analysis of MF transcriptional mechanisms that regulate molting, development, and reproduction in Crustacea.

## 2. Results

### 2.1. Taxonomic Distribution of the MEKRE93 Signaling and Co-Regulator Genes

BLAST sequence similarity searches of GenBank and transcriptome databases were used to identify MEKRE93 and co-regulator sequences in crustaceans. The sequences that were used in the initial queries are identified with an asterisk in the Supplementary Data Spreadsheet (see Section 4). The identity of each contig was verified through reciprocal BLAST searches and compiled in the Supplementary Data Spreadsheet. Phylogenetic trees were constructed and analyzed to validate and corroborate the BLAST results, as well as determine the evolutionary relationships of the MEKRE93 signaling and co-regulator genes in taxa across Clade Panarthropoda. Protein sequences were further characterized through multiple sequence alignments (MSAs) and annotations of conserved functional domains, motifs, and residues.

MEKRE93 signaling genes *Met*, *Src*, *Kr-h1*, and *E93* were identified across taxonomic groups within Phylum Arthropoda, while transcriptional co-regulators *CBP* and *CtBP* were identified across Clade Panarthropoda (Table 1). The absence of a sequence in a particular species reflects limitations of the transcriptome datasets (CrusTome, CrutsyBase, CrusTF, CAT, and GenBank) used for reciprocal BLAST searches and phylogenetic validation [84,85,87]. Sequences of all six genes were identified in Decapoda, Insecta, and Isopoda (Table 1). Five genes (*Met*, *Src*, *Kr-h1*, *CBP*, and *CtBP*) were identified in Branchiopoda. Four genes were identified in Amphipoda (*Met*, *Kr-h1*, *E93*, and *CtBP*), Copepoda (*Met, Kr-h1*, *CBP*, and *CtBP*), and Euphausiacea (*Kr-h1*, *E93*, *CBP*, and *CtBP*) (Table 1). Three genes (*E93*, *CBP*, and *CtBP*) were identified in Chelicerata. Two genes were identified in Branchiura (*Met* and *E93*) and Stomatopoda (*Kr-h1* and *CtBP*). *CtBP* was the most frequently detected under the filtering criteria, with 172 contigs identified in 116 species distributed across all taxa except Branchiura (Table 1). In crustacean species, single contig sequences for each of the six proteins were obtained, suggesting that crustacean genomes possess a single copy of each gene. In the Clade Multicrustacea, there was no evidence of isoforms generated by alternative mRNA splicing, although this requires confirmation by comparing mRNA and genomic sequences [64].

Contigs encoding MEKRE93 and co-regulator genes were identified in YO transcriptomes from four brachyuran species: the Chesapeake blue crab (*Callinectes sapidus*), the Dungeness crab (*Metacarcinus magister*), the green shore crab (*Carcinus maenas*), and the blackback land crab (*Gecarcinus lateralis*). In *G. lateralis*, complete coding sequences of *Gl-Met*, *Gl-Kr-h1*, *Gl-CBP*, and *Gl-CtBP* contigs and partial coding sequences of *Gl-Src* and *Gl-E93* contigs were identified in the YO transcriptome (Table 2; Supplemental Figures S1–S6). In *C. maenas*, complete coding sequences of *Cm-Kr-h1* and *Cm-E93* contigs and partial coding sequences of *Cm-Met*, *Cm-Src*, *Cm-CBP*, and *Cm-CtBP* contigs were identified in both YO and central nervous system (CNS) transcriptomes (Table 3; Supplemental Figures S7–S12).

### 2.2. Characterization of the MEKRE93 Signaling Genes

#### 2.2.1. Methoprene-Tolerant (Met)

**Figure 1:**
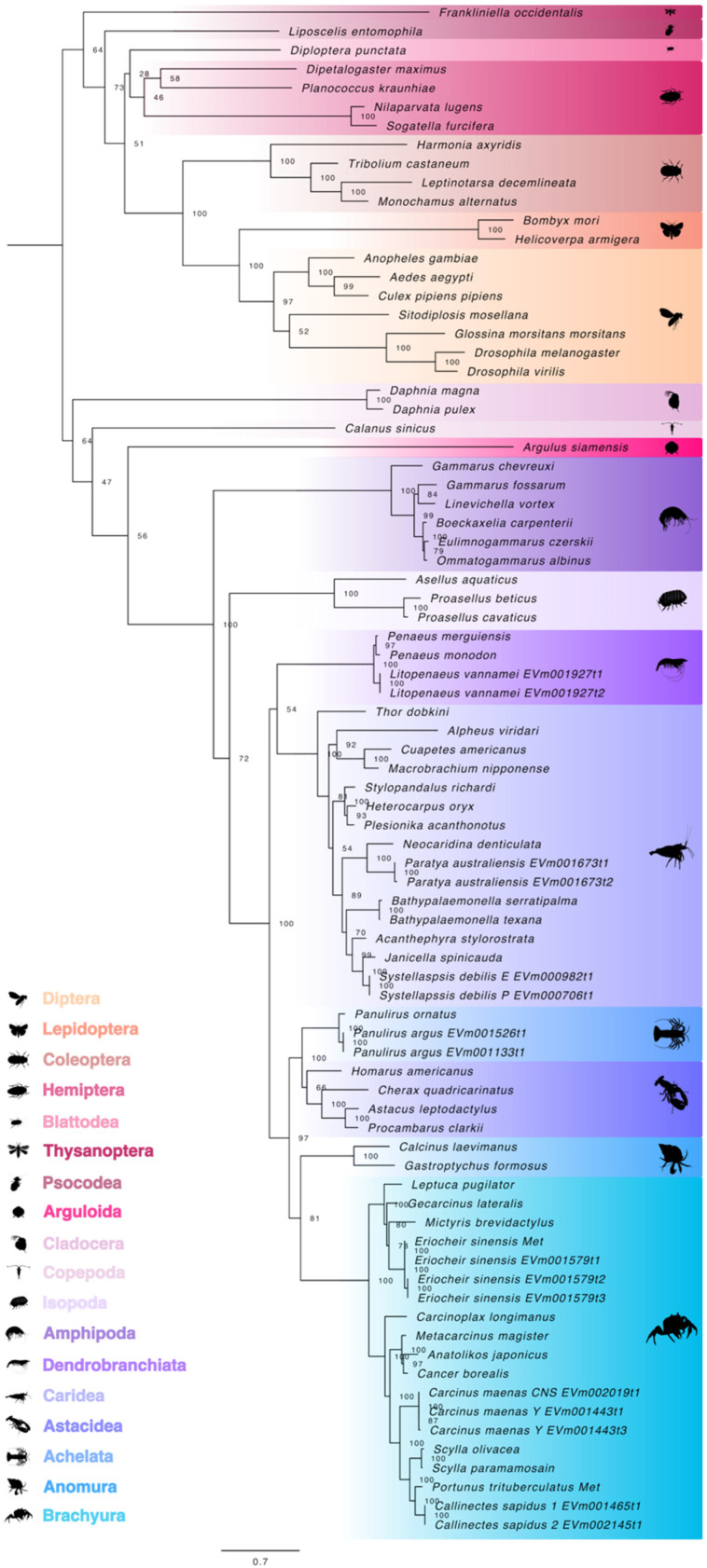
Phylogeny of pancrustacean Methoprene-tolerant (Met) proteins. The trimmed, midpoint rooted, maximum likelihood phylogenetic tree was constructed with IQ-TREE using the Bayesian information criterion (BIC) best-fit model JTT+F+I+R5. The confidence values at each branch point were determined with ultrafast bootstrap analysis (UFBoot = 1000). The scale bar for the branch lengths represents the estimated average number of substitutions per site as visualized in FigTree. Sequences and databases used in the analysis are provided in the Supplementary Data Spreadsheet. Species silhouettes were obtained from PhyloPic (http://phylopic.org; Supplementary Table S1).

To identify crustacean Met orthologs, Met protein sequences were used to construct a maximum likelihood tree. Phylogenetic analysis showed that the pancrustacean sequences were divided into two major groups corresponding to insects and crustaceans (Figure 1). Within each division, the sequences sorted along hierarchical taxonomic ranking of class, order, and infraorder. The Met proteins from the two *Daphnia* species, crustaceans within Clade Allotriocarida, were grouped with other crustaceans instead of with other members of Clade Allotriocarida (i.e., the insects) (Figure 1). The extracted Met sequence from the copepod *Calanus sinicus* was separated from other crustaceans within Class Malacostraca by the crustacean fish-louse (*Argulus siamensis*), a member of Superclass Oligostraca (Figure 1). The amphipod Met proteins were more phylogenetically divergent when compared to the other malacostracan orders, such as isopods and decapods (Figure 1). Decapod Met sequences segregated into two groups with one group containing lobster (e.g., Achelata and Astacidea) and crab (e.g., Anomura and Brachyura) infraorders and the other group containing the shrimp infraorders (e.g., Caridea and Dendrobranchiata) (Figure 1).

**Figure 2:**
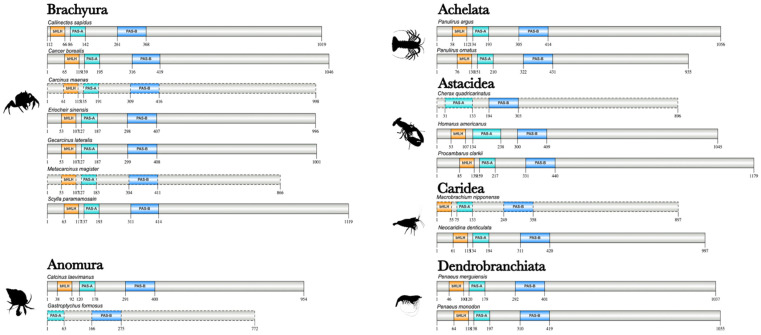
Domain organization of decapod Methoprene-tolerant (Met) proteins. Domains were identified with the NCBI CD search tool and visualized with IBS 2.0. Dashed outlines indicate partial sequences. The N-terminal region contained the basic helix-loop-helix (bHLH) DNA-binding domain, followed by the PAS-A and PAS-B domains. Information for the sequences is provided in the Supplementary Data Spreadsheet. Species silhouettes were obtained from PhyloPic (http://phylopic.org; Supplementary Table S1).

The domain organization of Met proteins was analyzed across 18 decapod species (Figure 2). The N-terminal region had the same arrangement of three conserved domains: an N-terminal bHLH domain followed by two PAS domains (PAS-A and PAS-B), respectively (Figure 2). Decapod and amphipod Met orthologs were ~1K amino acids in length, whereas the cladoceran and isopod Met orthologs were shorter (~690 amino acids and 766 amino acids, respectively; Supplemental Figure S13). The lengths of decapod Met sequences were double that in some beetle species (Order Coleoptera) and the domestic silk moth (*Bombyx mori*; Order Lepidoptera), while similar in length to some hemipterans (Supplemental Figure S14).

**Figure 3:**
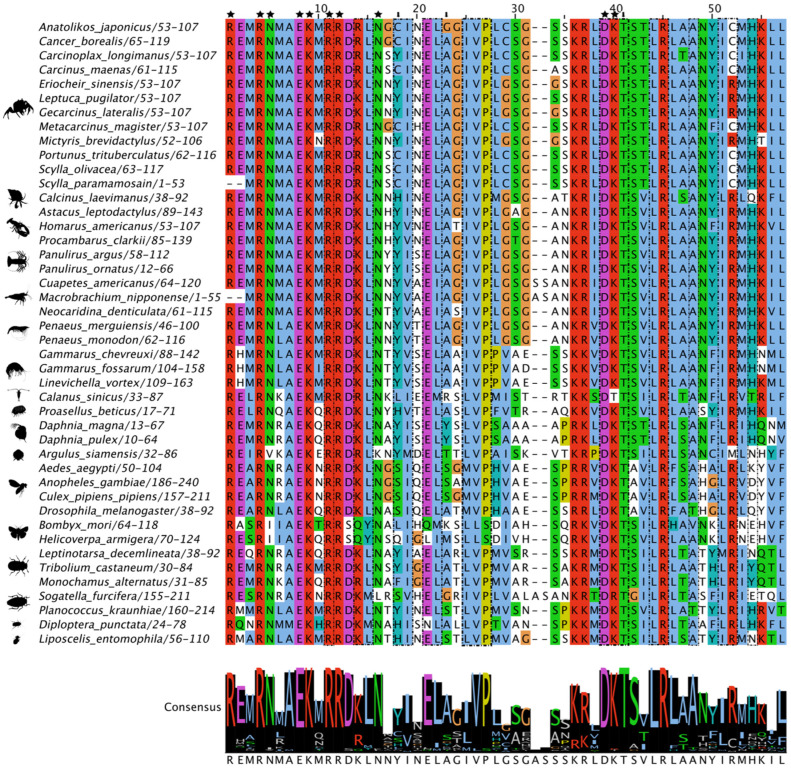
Multiple sequence alignment (MSA) of pancrustacean Methoprene-tolerant (Met) basic helix-loop-helix (bHLH) domain. The sequences were aligned using the Mafft EINSI parameters, trimmed with ClipKIT, and visualized through Jalview following default Clustal coloring. The consensus sequence is illustrated as a logo schematic. Conserved amino acids involved with DNA binding and dimer interfaces are indicated by black stars and by dashed lines, respectively. Phosphorylation site reported in *Aedes aegypti* is indicated by the black arrow [91]. Sequences and databases used in the analysis are provided in the Supplementary Data Spreadsheet. Species silhouettes were obtained from PhyloPic (http://phylopic.org; Supplementary Table S1).

**Figure 4:**
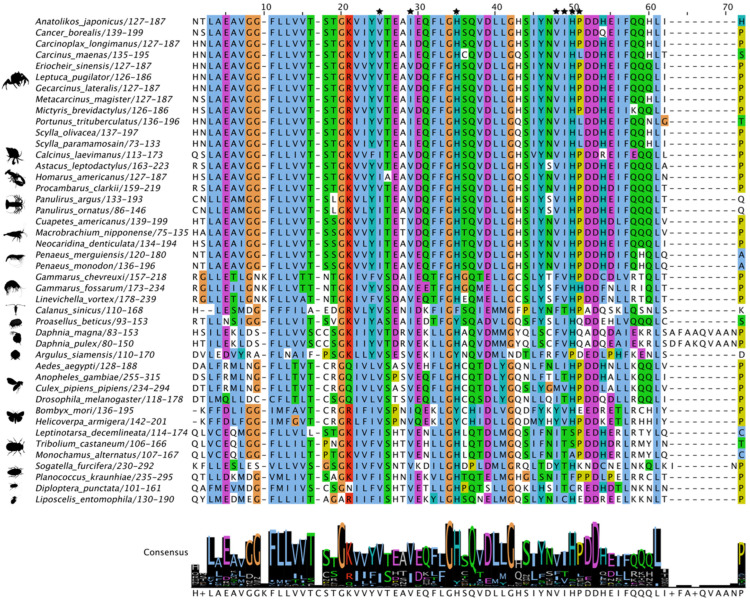
Multiple sequence alignment (MSA) of pancrustacean Methoprene-tolerant (Met) PAS-A domain. The sequences were aligned using the Mafft EINSI parameters, trimmed with ClipKIT, and visualized through Jalview following default Clustal coloring. The consensus sequence is illustrated as a logo schematic. Conserved amino acids as active sites are indicated by black stars. Sequences and databases used in the analysis provided in the Supplementary Data Spreadsheet. Species silhouettes were obtained from PhyloPic (http://phylopic.org; Supplementary Table S1).

**Figure 5:**
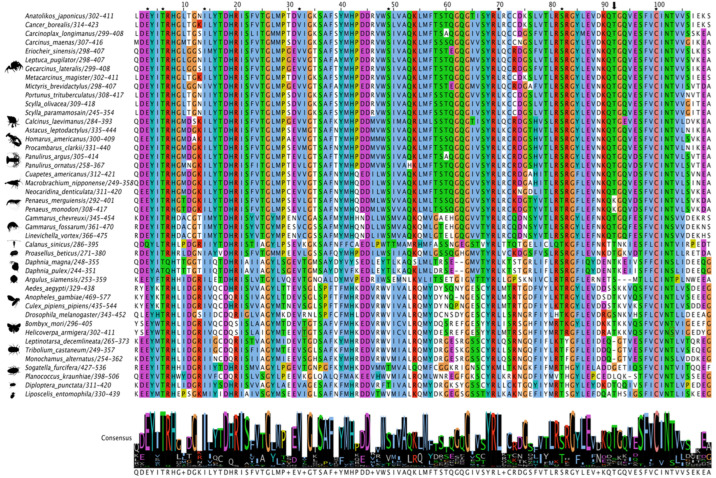
Multiple sequence alignment (MSA) of pancrustacean Methoprene-tolerant (Met) PAS-B domain. The sequences were aligned using the Mafft EINSI parameters, trimmed with ClipKIT, and visualized through Jalview following default Clustal coloring. The consensus sequence is illustrated as a logo schematic. The reported positions of eight amino acids involved in JH binding in insects are indicated by the black stars. Phosphorylation site reported in *Helicoverpa armigera* [93] is indicated by the black arrow along with putative sites boxed in red (positions #33, #66, #72, and #103). Sequences and databases used in the analysis are provided in the Supplementary Data Spreadsheet. Species silhouettes were obtained from PhyloPic (http://phylopic.org; Supplementary Table S1).

While the C-terminal region was more variable, MSAs of Met sequences showed that the three domains in the N-terminus were highly conserved. The 55-amino acid bHLH domain had high amino acid identity, including 10 residues involved in DNA binding (Figure 3). In decapods, the PAS-A domain had high sequence identities/similarities, including the seven active site residues (Figure 4; reference positions #25, #29, #35, #48, #49, #50, and #51). Seven of the eight residues in the PAS-B domain involved in JH binding in insect Met were identical in all the decapod sequences (Figure 5; reference positions #3, #6, #14, #32, #49, #70, and #100). Two penaeid species, *Penaeus monodon* and *P. merguiensis*, had a threonine at position #82, while all the other decapod species had a serine (Figure 5). Putative phosphorylation sites were identified in different regions of Met. In the bHLH domain, the serine at position #23 was only conserved in three mosquito species (*A. aegypti*, *Anopheles gambiae*, and *Culex pipiens pipiens*), the Japanese mealybug (*Planococcus krauhiae*), and the grain psocid (*Lipscelis entomophila*) (Figure 3) [91]. The serine at *P. kraunhiae* position #18 was identified in *D. melanogaster* as a critical phosphorylation site and exhibited high conservation among the dipteran species, but not across other pancrustacean species (Figure 3) [92]. The threonine at position #41, which is an additional known phosphorylation site in *D. melanogaster* [92], was highly conserved across the pancrustaceans. In the PAS-B domain, a threonine at position #92, as reported in the cotton bollworm (*Helicoverpa armigera*) [93], was conserved in decapods, except for penaeid shrimp, in which a lysine replaced the threonine (Figure 5). The threonine at position #92 was absent in coleopterans and hemipterans, while *A. aegypti*, *D. melanogaster*, and the fish louse (*A. siamensis*) had a serine substitution (Figure 5).

#### 2.2.2. Steroid Receptor Coactivator (Src)

**Figure 6:**
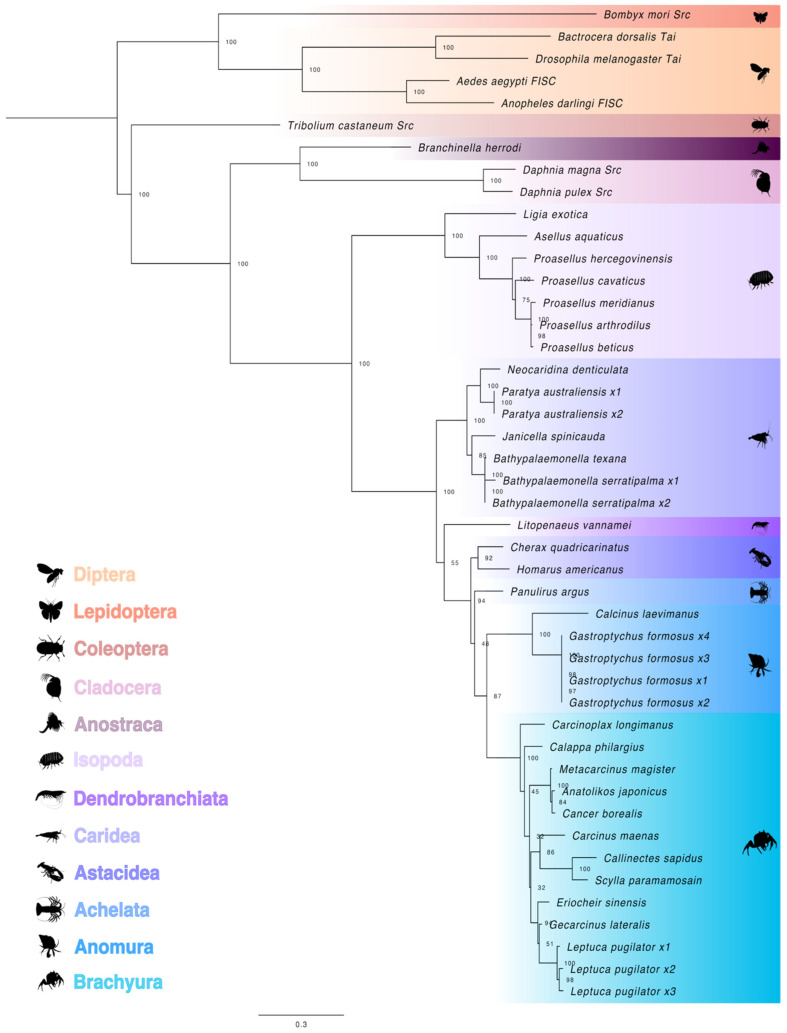
Phylogeny of pancrustacean Steroid receptor coactivator (Src) proteins. The trimmed maximum likelihood phylogenetic tree was constructed with IQ-TREE using the Bayesian information criterion (BIC) best-fit model JTT+F+I+G4. The confidence values at each branch point were determined with ultrafast bootstrap analysis (UFBoot = 1000). The scale bar for the branch lengths represents the estimated average number of substitutions per site as visualized in FigTree. Sequences and databases used in the analysis are provided in the Supplementary Data Spreadsheet. Species silhouettes were obtained from PhyloPic (http://phylopic.org; Supplementary Table S1).

Src proteins were divided into the insect and the crustacean groups with subclades organized along lower taxonomic hierarchy, as shown in the strongly supported maximum likelihood phylogenetic tree (Figure 6). In crustaceans, the Src proteins diverged between Class Branchiopoda (Orders Cladocera and Anostraca) and Class Malacostraca. No Src orthologs were identified in amphipods. Multiple Src variants were identified in some decapod species, such as *Paratya australiensis*, *Bathypalaemonella serratipalma*, *Gastroptychus formosus*, and *Leptuca pugilator* (Figure 6; Supplementary Data). A Src sequence was identified in one penaeid shrimp (*L. vannamei*) (Figure 6; Supplementary Data). Decapod Src sequences were further divided between the crab infraorders (Anomura and Brachyura) and the lobster and shrimp infraorders (Figure 6).

**Figure 7:**
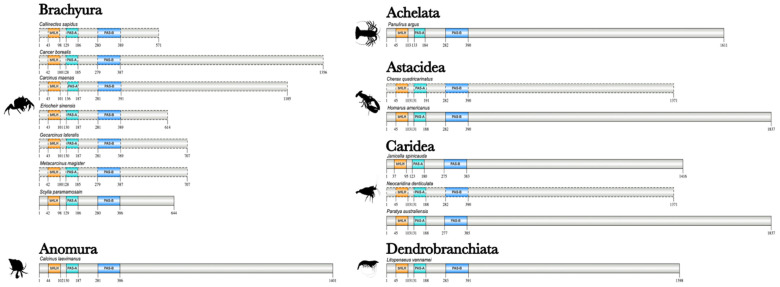
Domain organization of decapod Steroid receptor coactivator (Src) proteins. Domains were identified with the NCBI CD search tool and visualized with IBS 2.0. Dashed outlines indicate partial sequences. The N-terminal region contained the basic helix-loop-helix (bHLH) DNA-binding domain, followed by the PAS-A and PAS-B domains. Information for the sequences is provided in the Supplementary Data Spreadsheet. Species silhouettes were obtained from PhyloPic (http://phylopic.org; Supplementary Table S1).

The domain organization of the Src proteins, with the N-terminal region encompassing the bHLH, PAS-A, and PAS-B domains, was conserved among the 14 decapod species (Figure 7). The Src sequences showed high sequence identity in the three functional domains; the C-terminal region varied in length and amino acid sequence (Figure 7; Supplemental Figures S15 and S16; Supplementary Data).

#### 2.2.3. Krüppel Homolog 1 (Kr-h1)

**Figure 8:**
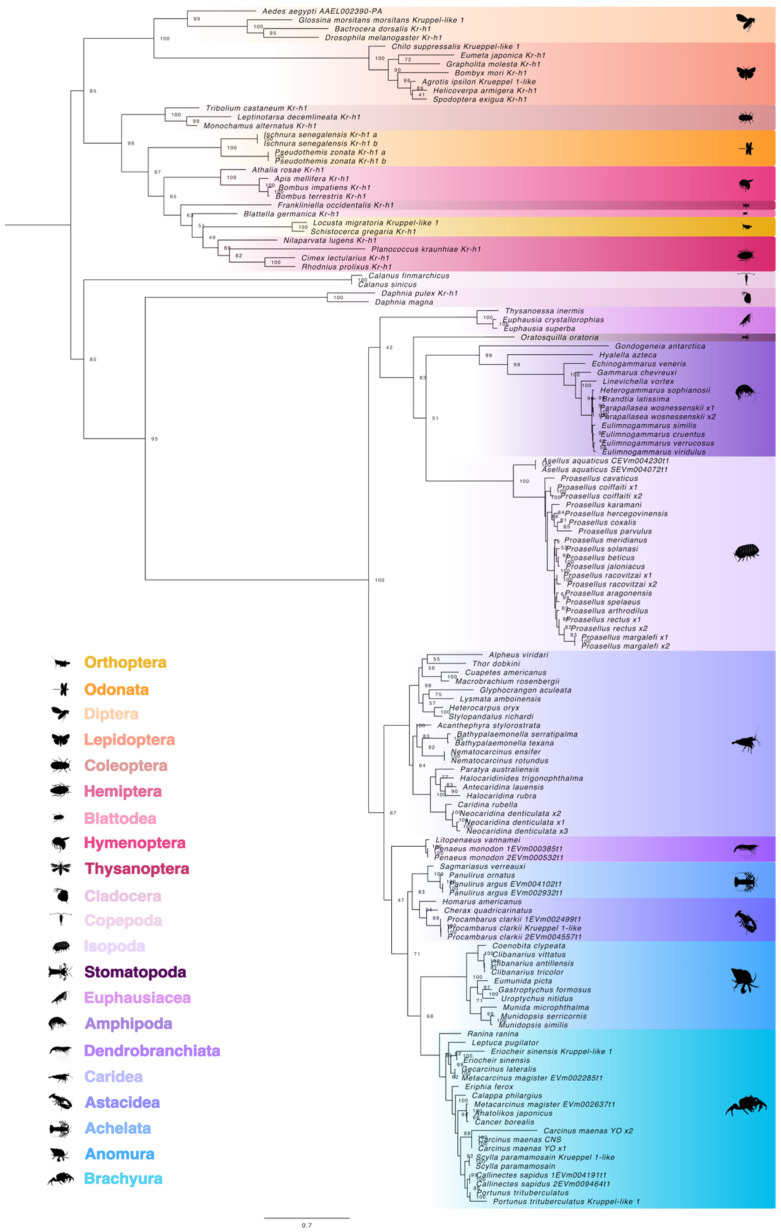
Phylogeny of pancrustacean Krüppel homolog 1 (Kr-h1) proteins. The trimmed maximum likelihood phylogenetic tree was constructed with IQ-TREE using the Bayesian information criterion (BIC) best-fit model JTT+I+I+R5. The confidence values at each branch point were determined with ultrafast bootstrap analysis (UFBoot = 1000). The scale bar for the branch lengths represents the estimated average number of substitutions per site as visualized in FigTree. Sequences and databases used in the analysis are provided in the Supplementary Data Spreadsheet. Species silhouettes were obtained from PhyloPic (http://phylopic.org; Supplementary Table S1).

Maximum-likelihood phylogenetic analysis of the maximum likelihood tree of Kr-h1 sequences separated the pancrustacean proteins into insect and crustacean groups (Figure 8). Apart from the copepod species, further division of Kr-h1 orthologs followed taxonomic hierarchy, which was supported by strong bootstrap values. The copepods (members of Clade Multicrustacea) were positioned adjacent to Clade Allotriocarida members (e.g., insects and *Daphnia* sp.) instead of clustering with other orders within this clade, including Euphausiacea and Decapoda (Figure 8). Within the Multicrustacea, decapod Kr-h1 sequences were segregated from the Kr-h1 sequences in Orders Euphausiacea, Amphipoda, and Isopoda, as indicated by highly supported, long branch lengths (Figure 8). Insect Kr-h1 orthologs clustered closely, with short branch lengths across taxonomic orders, whereas crustacean orthologs exhibited longer branch lengths between the different taxonomic groups (Figure 8).

**Figure 9:**
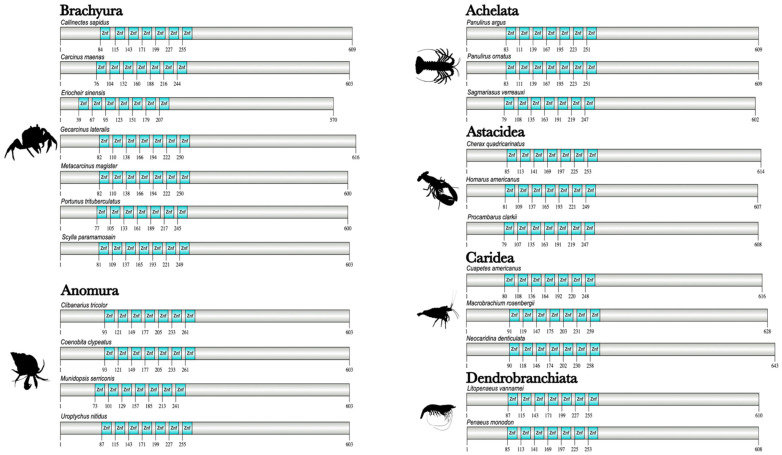
Domain organization of decapod Krüppel homolog 1 (Kr-h1) proteins. Domains were identified with the NCBI CD search tool and visualized with IBS 2.0. Dashed outlines indicate partial sequences. The N-terminal region contained seven zinc finger (Znf) repeats in the DNA-binding domain. Information for the sequences is provided in the Supplementary Data Spreadsheet. Species silhouettes were obtained from PhyloPic (http://phylopic.org; Supplementary Table S1).

**Figure 10:**
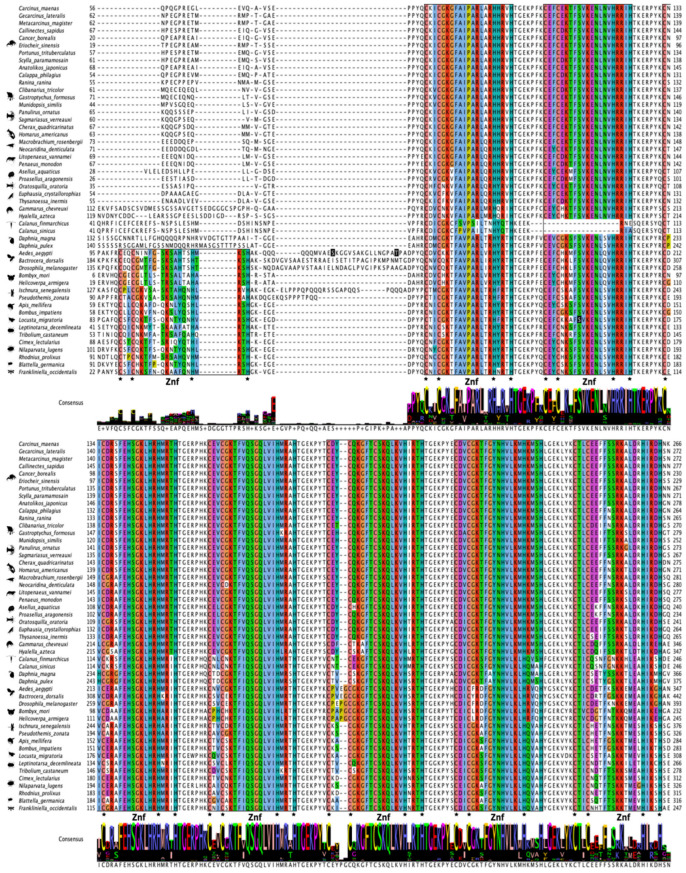
Multiple sequence alignment (MSA) of pancrustacean Krüppel homolog 1 (Kr-h1) proteins. The sequences were aligned using the Mafft EINSI parameters, trimmed with ClipKIT, and visualized through Jalview. The consensus sequence is illustrated as a logo schematic. The C_2_H_2_ zinc fingers are annotated following default Clustal coloring with the two Cys and His residues indicated by black stars. Sequences and databases used in the analysis are provided in the Supplementary Data Spreadsheet. Residues highlighted in black are functionally annotated post-translational modifications (PTM) (e.g., phosphorylation and acetylation) sites; refer to Figure S19 for additional PTM sites in the C-terminus identified in *Aedes aegypti* [91]. Species silhouettes were obtained from PhyloPic (http://phylopic.org; Supplementary Table S1).

The domain organization of decapod Kr-h1 orthologs was highly conserved in 22 decapod species (Figure 9). The N-terminal region had a series of DNA-binding domains consisting of seven C_2_H_2_ Znf repeats of ~21 amino acids in length (Figure 9). MSA analysis of pancrustacean Kr-h1 orthologs showed high sequence identity of the C_2_H_2_ Znf repeats, with insect Kr-h1 having eight repeats and crustacean Kr-h1 having seven repeats (Figure 10; Supplementary Figures S17 and S18). Although grouped within Clade Allotriocarida along with the insects, the *Daphnia* Kr-h1 sequences (781–860 amino acids) had seven C_2_H_2_ Znf repeats as in other crustaceans but was similar in length (791 amino acids) to *D. melanogaster* Kr-h1 (Supplementary Figures S17 and S18). Thus, the difference in the number of C_2_H_2_ Znf repeats was a distinguishing feature between insect and crustacean Kr-h1 orthologs.

Post-translational modification sites, such as phosphorylation and acetylation, that were identified in the yellow fever mosquito (*A. aegypti*; [91]) were not conserved in crustacean Kr-h1 sequences (Figure 10; Supplementary Figure S18). Only Kr-h1 sequences in American lobster (*Homarus americanus*) and the krill *Euphausia crystallorophias* shared a conserved serine in the C-terminal region that was identified by NetPhos 3.1 as a potential phosphorylation site (Supplementary Figure S18). Pancrustacean species, except for copepods, contained a conserved KxFSVKNLxVHRR sequence within the second (in crustacean species) or third (in insect species) Znf repeat, in which the serine is phosphorylated [13] (Figure 10; Ser154 in locust Kr-h1 highlighted in black).

#### 2.2.4. E93

**Figure 11:**
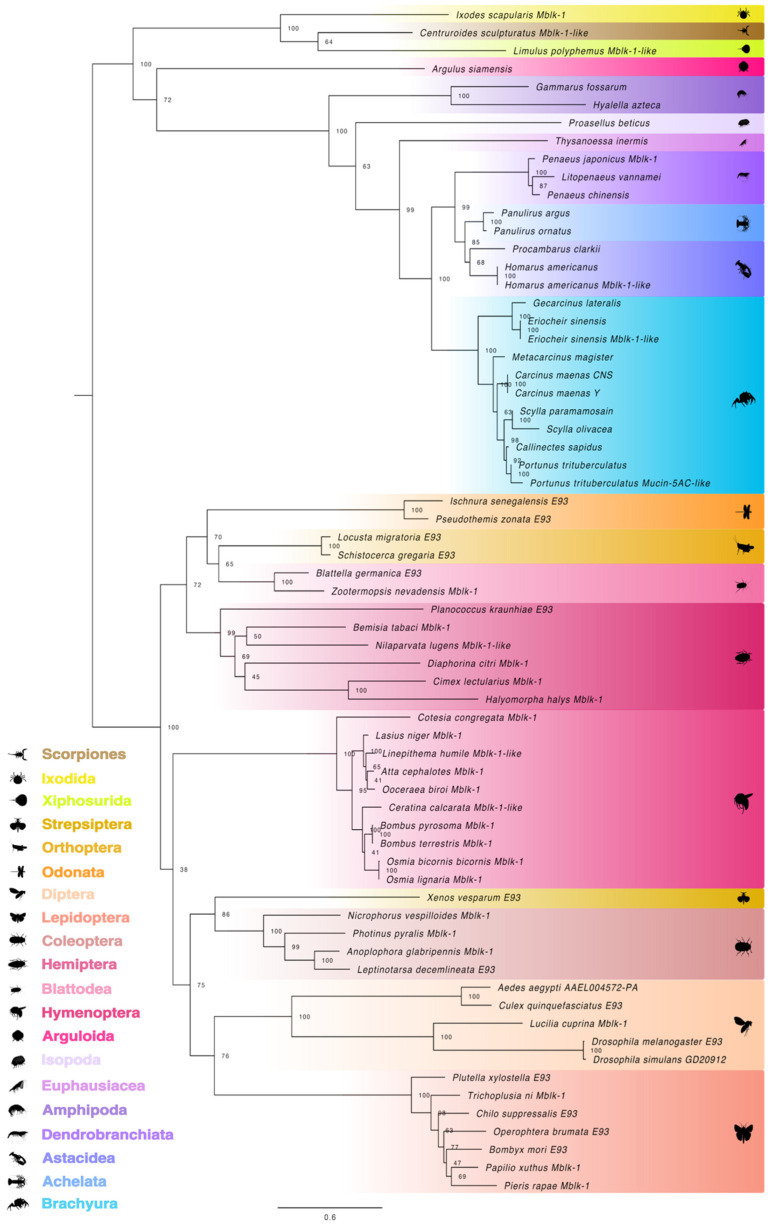
Phylogeny of arthropod E93 and Mushroom body large-type Kenyon cell-protein 1 (Mblk-1) proteins. The trimmed (midpoint rooted) maximum likelihood phylogenetic tree was constructed with IQ-TREE using the Bayesian information criterion (BIC) best-fit model JTT+F+I+R5. The confidence values at each branch point were determined with ultrafast bootstrap analysis (UFBoot = 1000). The scale bar for the branch lengths represents the estimated average number of substitutions per site as visualized in FigTree. Sequences and databases used in the analysis are provided in the Supplementary Data Spreadsheet. Species silhouettes were obtained from PhyloPic (http://phylopic.org; Supplementary Table S1).

Annotated E93 amino acid sequences were used to construct a maximum likelihood phylogenetic tree that included the hypothesized E93 orthologs annotated as Mushroom body large-type Kenyon cell protein-1 (Mblk-1) [94]. With chelicerate species serving as the outgroup, phylogenetic analysis showed segregation of pancrustacean E93/Mblk-1 proteins into insect and crustacean groups; no E93 orthologs were identified in *Daphnia* and copepods (Figure 11). Within each group, the E93 orthologs segregated according to taxonomic hierarchy. In the crustacean fish-louse (*A. siamensis*)*,* a member of Superclass Oligostraca, the Mblk-1/E93 sequence was positioned between chelicerate and malacostracan E93 sequences (Figure 11). Within Class Malacostraca, Mblk-1/E93 sequences were partitioned into amphipod, isopod, krill, shrimp/prawn, lobster/crayfish, and crab subgroups (Figure 11). Insect E93/Mblk-1 proteins clustered according to taxonomic rank, supporting their classification as orthologs (Figure 11).

**Figure 12:**
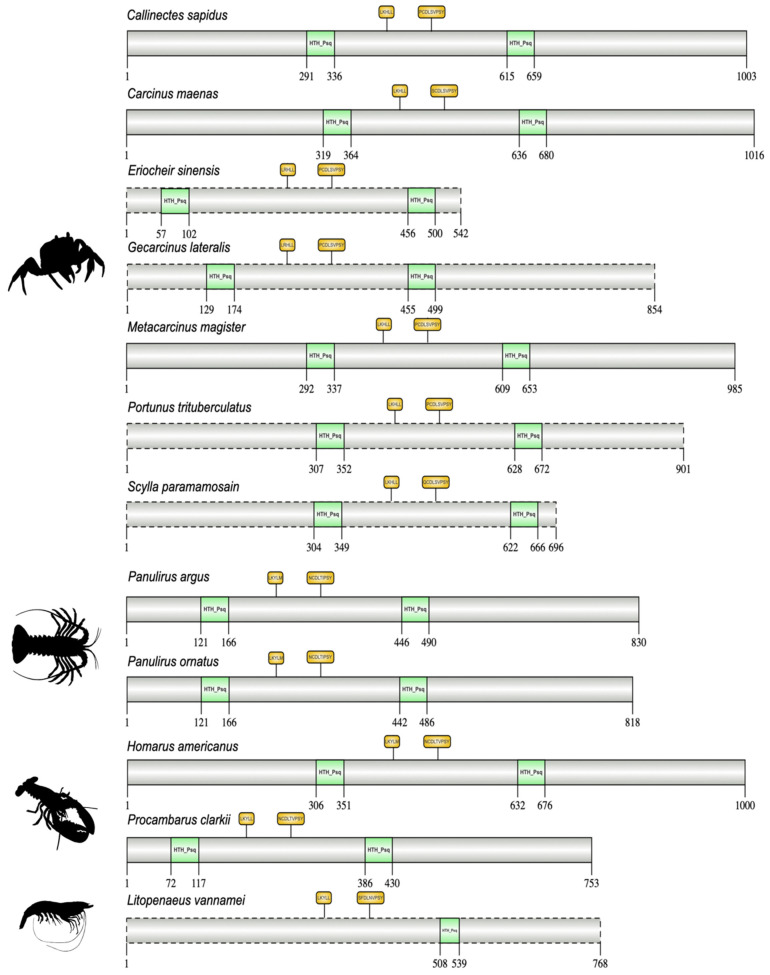
Domain organization of decapod E93/Mblk-1 proteins. Domains were identified with the NCBI CD search tool and visualized with IBS 2.0. Dashed outlines indicate partial sequences. Proteins, except Lv-E93, contained two helix-turn-helix, pipsqueak (HTH_Psq) DNA-binding domains. A nuclear receptor interaction sequence (e.g., LxxLL or LLxxL motif) and the C-terminal-binding protein (CtBP) interaction sequence (e.g., PxDLSVPS motif) is contained within the two HTH_Psq domains. Information for the sequences is provided in the Supplementary Data Spreadsheet. Species silhouettes were obtained from PhyloPic (http://phylopic.org; Supplementary Table S1).

Decapod E93/Mblk-1 sequences had two HTH_Psq domains, except for *L. vannamei* E93, which contained only one HTH_Psq domain (Figure 12). Aside from the HTH_Psq domains, the pancrustacean E93/Mblk-1 sequences varied in length and composition even within taxonomic groups (Figure 12; Supplementary Figures S20 and S21). Two interaction sequences were identified between the two HTH_Psq domains in pancrustacean E93/Mblk-1 orthologs (Figure 12; Supplemental Figure S22). The nuclear receptor interaction sequence (e.g., LxxLL or LLxxL motif) was conserved among brachyurans, as well as other crustaceans and the insect relatives (Figure 12; Supplementary Figure S22) [95]. Downstream of the nuclear receptor interaction sequence was the transcriptional co-repressor C-terminal binding protein (CtBP) interaction sequence (e.g., PxDLSVPS motif; Figure 12 and Supplementary Figure S22) [96]. The CtBP interaction sequence was highly conserved across brachyurans and, more broadly, among pancrustaceans (Supplementary Figure S22). Crustacean E93/Mblk-1 sequences possessed a conserved serine, which is a potential protein kinase C (PKC) phosphorylation site in most insect taxa (Supplementary Figure S22) [94].

### 2.3. Transcriptional Co-Regulators

#### 2.3.1. CREB-Binding Protein (CBP)

**Figure 13:**
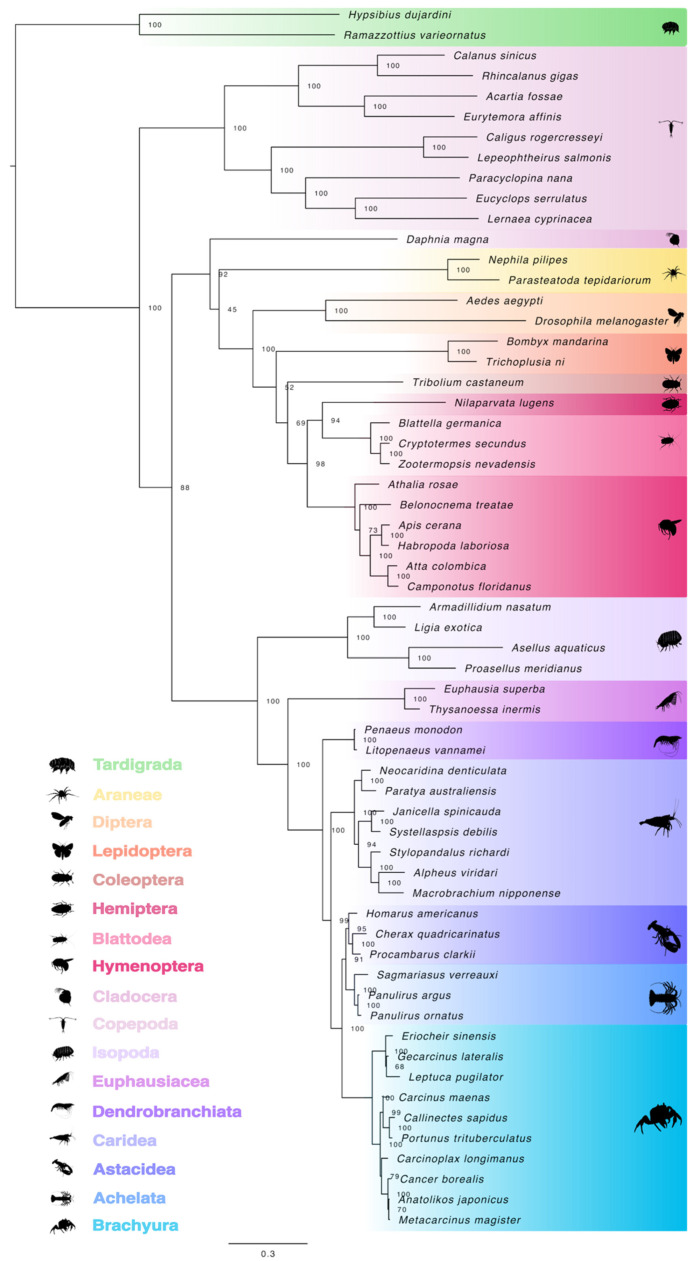
Phylogeny of Panarthropoda CREB-binding proteins (CBPs). The trimmed maximum likelihood phylogenetic tree was constructed with IQ-TREE using the Bayesian information criterion (BIC) best-fit model JTT+F+I+I+R5. The confidence values at each branch point were determined with ultrafast bootstrap analysis (UFBoot = 1000). The scale bar for the branch lengths represents the estimated average number of substitutions per site as visualized in FigTree. Sequences and databases used in the analysis are provided in the Supplementary Data Spreadsheet on the online repository. Species silhouettes were obtained from PhyloPic (http://phylopic.org; Supplementary Table S1).

A maximum likelihood phylogenetic tree was constructed and rooted with tardigrade CBP sequences to identify crustacean CBP orthologs. The three major clades did not always follow taxonomic groupings, as the pancrustacean sequences did not group together. Copepod CBPs formed a separate group from other malacostracans, such as the isopods, krill, and decapods (Figure 13). At the copepod branchpoint, the Pancrustacea partitioned into insect and crustacean groups with *Daphnia* positioned with the insects, as both are members of Clade Allotriocarida. Two spider CBPs were positioned between *Daphnia* and the insect orders (Figure 13). Malacostracan CBP sequences followed taxonomic order, with decapod CBPs grouping according to infraorder hierarchy (Figure 13).

**Figure 14:**
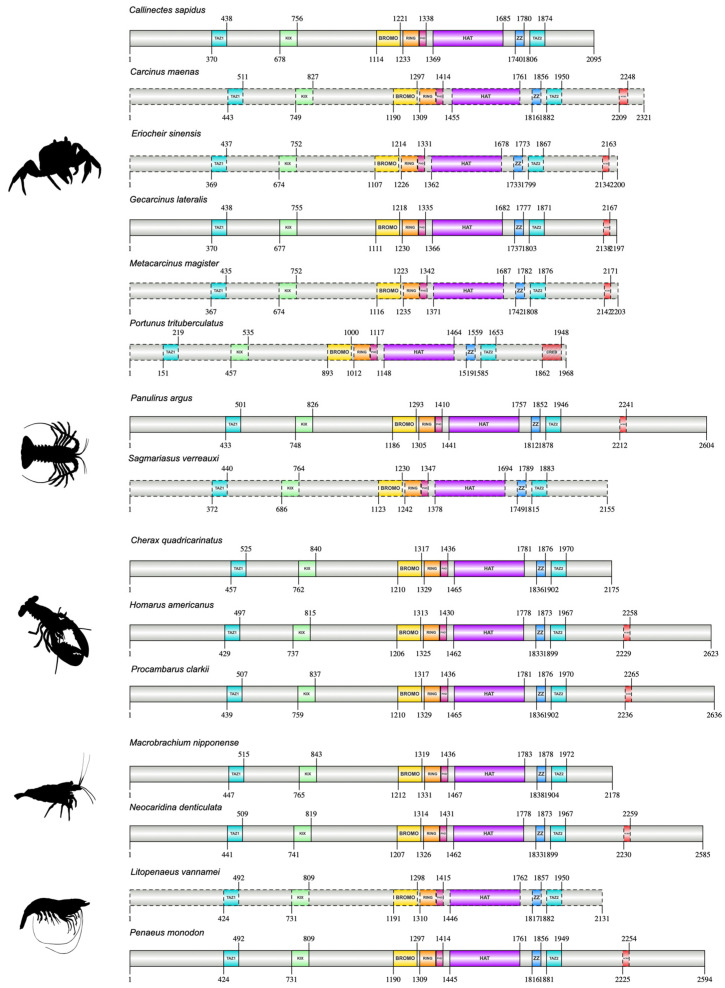
Domain organization of decapod CBPs. Domains were identified with the NCBI CD search tool and visualized with IBS 2.0. Dashed outlines indicate partial sequences. CBPs contained the Transcription Adaptor Zinc Finger (TAZ1), Kinase-inducible domain (KID) interacting domain (KIX), Bromodomain (BROMO), Really Interesting New Gene (RING), Plant Homeodomain (PHD), Histone acetyltransferase (HAT), ZZ zinc finger (ZZ), and TAZ2 domains. Coactivator binding sites were contained in the nuclear receptor co-activator binding domain (NCBD) within the CREB binding regions (CREB). Information for the sequences is provided in the Supplementary Data Spreadsheet. Species silhouettes were obtained from PhyloPic (http://phylopic.org; Supplementary Table S1).

CBPs of 15 decapod species were over 2K amino acids in length and had the same domain organization as annotated CBPs (Figure 14; Supplementary Figures S23 and S24). CBPs contained three cysteine/histidine-rich regions flanked by transactivation domains (e.g., transcriptional adaptor zinc finger or TAZ domains) at the N- and C-termini and also harbored the series of functional domains including the BROMO domain; the CH2 domain (i.e., with the BROMO, PHD, and HAT subdomains), CH3 domain (i.e., with the ZZ and TAZ2), and the nuclear co-activator binding domain (NCBD) (Figure 14; Supplementary Figures S23 and S24). Across infraorders, the lengths varied after the NCBD, with the HAT domain the longest in length. Within Infraorder Astacidea, the CBP ortholog was 2623 amino acids in *H. americanus*, 2636 amino acids in *Procambarus clarkii*, and 2175 amino acids *Cherax quadricarinatus* (Figure 14). The CBP orthologs within Infraorder Caridea, specifically in *Macrobrachium nipponense* and *N. denticulata* were 2178 and 2585 amino acids in length, respectively (Figure 14). The majority of crustacean CBP orthologs, except *Daphnia*, were shorter than the dipteran CBPs (Supplementary Figures S23 and S24).

#### 2.3.2. C-Terminal-Binding Protein (CtBP)

**Figure 15:**
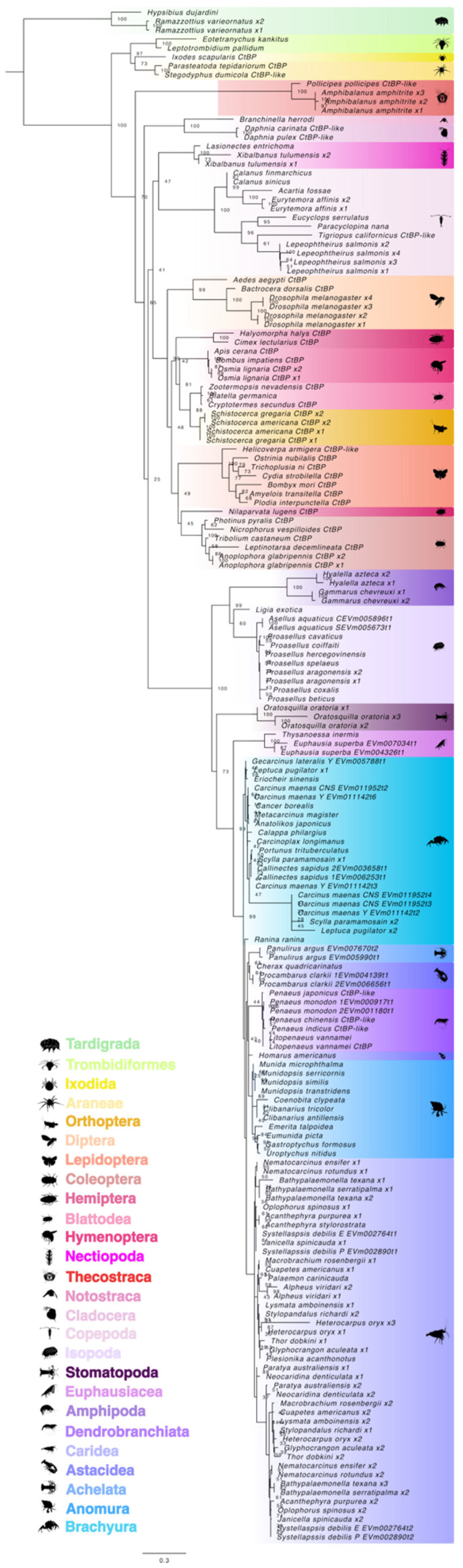
Phylogeny of C-terminus-binding proteins (CtBPs) in Clade Panarthropoda. The trimmed maximum likelihood phylogenetic tree was constructed with IQ-TREE using the Bayesian information criterion (BIC) best-fit model Dayhoff+I+R4. The confidence values at each branch point were determined with ultrafast bootstrap analysis (UFBoot = 1000). The scale bar for the branch lengths represents the estimated average number of substitutions per site as visualized in FigTree. Sequences and databases used in the analysis are provided in the Supplementary Data Spreadsheet. Species silhouettes were obtained from PhyloPic (http://phylopic.org; Supplementary Table S1).

A maximum likelihood phylogenetic tree was constructed to identify crustacean CtBP orthologs. Phylogenetic analysis showed a distinct separation between arthropods and tardigrades, which served as the outgroup (Figure 15). Furthermore, the division between the chelicerates and mandibulates was strongly supported by bootstrap values, except for the barnacle species (Order Thecostraca). Class Branchiopoda (Orders Cladocera and Notostraca) CtBPs were more closely related to other crustaceans (e.g., Nectiopoda, Copepoda), even though they are classified with insects in Clade Allotriocarida (Figure 15). Within Class Malacostraca, CtBP sequences followed taxonomic order and infraorder hierarchy. Many caridean shrimp species possessed long and short CtBP transcripts, whereas only a single CtBP ortholog was identified in the other decapod infraorders (Brachyura, Anomura, Achelata, Astacidea, and Dendrobranchiata) (Figure 15).

**Figure 16:**
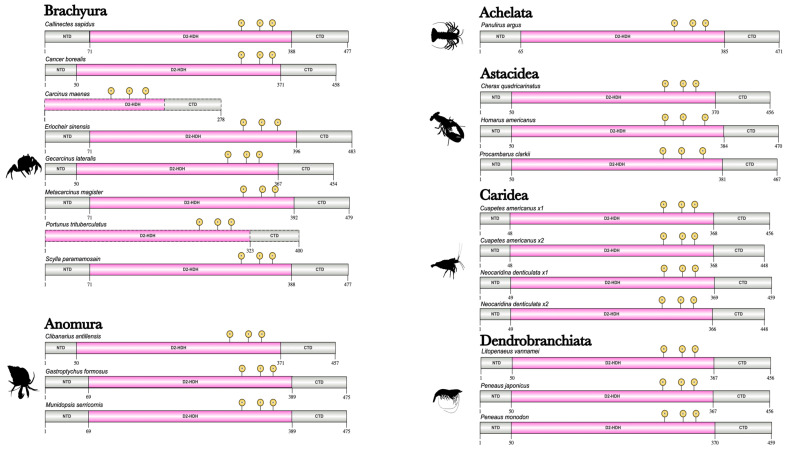
Domain organization of decapod CtBPs. Domains were identified with the NCBI CD search tool and visualized with IBS 2.0. Dashed outlines indicate partial sequences. The D-isomer specific 2-hydroxyacid dehydrogenase (D2-HDH) domain, flanked by the N-terminal domain (NTD) and the C-terminal domain (CTD), contains the R-E-H catalytic residue sites (indicated in yellow). Information for the sequences is provided in the Supplementary Data Spreadsheet. Species silhouettes were obtained from PhyloPic (http://phylopic.org; Supplementary Table S1).

**Figure 17:**
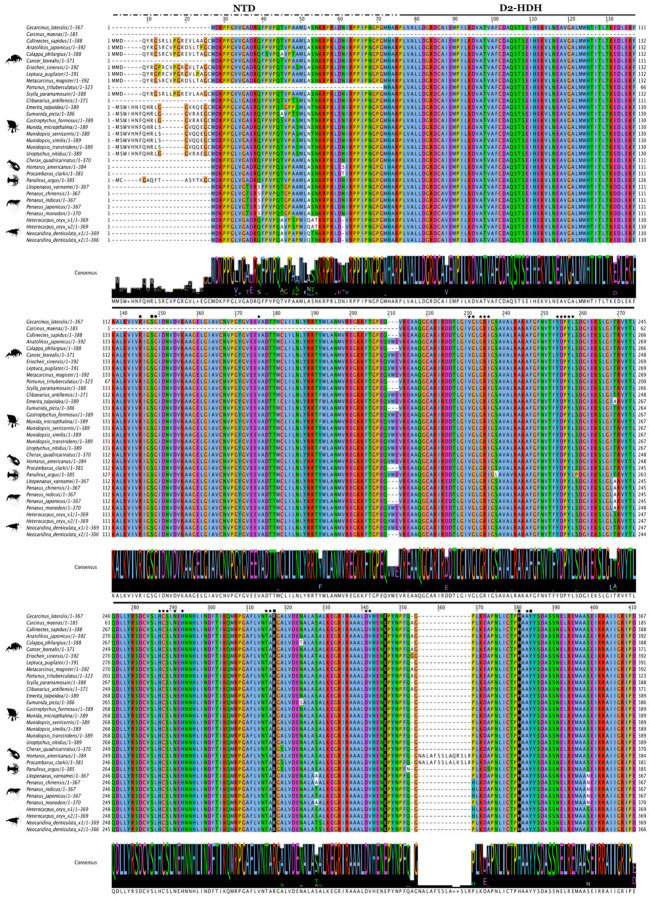
Multiple sequence alignment (MSA) of decapod crustacean CtBP N-terminal region. The N-terminal domain (NTD), indicated by the overhead dashed line, precedes and flanks the D-isomer specific 2-hydroxyacid dehydrogenase domain (D2-HDH), shown by the overhead solid line. The sequences were aligned using the Mafft EINSI parameters, trimmed with ClipKIT, and visualized through Jalview following default Clustal coloring. The consensus sequence is illustrated as a logo schematic. The D2-HDH domain contains the Rossmann fold along with the diagnostic catalytic triad of Arginine (R), Glutamic Acid (E), and Histidine (H) (highlighted in black). Black stars indicate conserved amino acids involved in NAD-binding, black circles indicate ligand-binding, and black rectangles show NAD/ligand-binding. Sequences and databases used in the analysis are archived in the Supplementary Data Spreadsheet. Species silhouettes were obtained from PhyloPic (http://phylopic.org; Supplementary Table S1).

**Figure 18:**
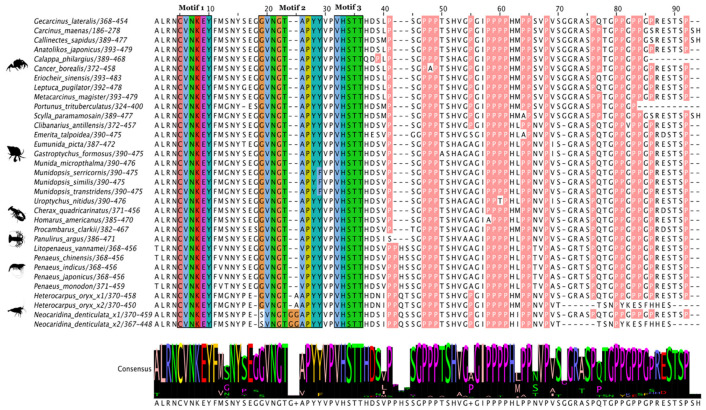
Multiple sequence alignment (MSA) of the disordered C-terminal domain (CTD) of decapod crustacean CtBPs. The sequences were aligned using the Mafft EINSI parameters, trimmed with ClipKIT, and visualized through Jalview. The consensus sequence is illustrated as a logo schematic. As visualized following default Clustal coloring, the CTD begins with the CVNKEY motif and is followed by the central block motif (GVNGTAPYY in brachyurans) and later the VHSTT motif. A proline (P) rich region (shown in peach) subsequently succeeds these motifs. Sequences and databases used in the analysis are archived in the Supplementary Data Spreadsheet. Species silhouettes were obtained from PhyloPic (http://phylopic.org; Supplementary Table S1).

CtBP across Clade Panarthropoda consisted of three major domains: the N-terminal domain (NTD), the D2-HDH domain, and the C-terminal domain (CTD) (Figure 16; Supplementary Figures S25 and S26). The NTD, which contained multiple putative phosphorylation and SUMOylation sites showed high sequence conservation among decapods, pancrustaceans, and even Panarthropoda, which was consistent with reports across all bilaterians [64,65,66] (Figure 17; Supplementary Figures S27 and S28). Further analysis of the CtBP sequences showed that the D2-HDH domain was also highly conserved and contained the diagnostic REH catalytic triad (Figure 17; Supplementary Figures S27 and S28). By contrast, the CTD was a proline- and glycine-rich structurally disordered region that contained putative phosphorylation and SUMOylation sites [66] (Figure 18; Supplementary Figures S27 and S28). In amphipods and the tadpole shrimp, polyalanine stretches replaced the proline repeats, which also occurred in CTDs in tardigrades and *D. melanogaster* (Supplementary Figure S27). The CTD contained the conserved the CVNKEY and NGGYY/GLNG-–YY central block and AHSTT motifs with polyproline repeats following the central block (Table 4; Figure 18; Supplementary Figures S27 and S28). Only barnacle species had the ancestral SEVH motif (Table 4; Supplementary Figure S28).

## 3. Discussion

The CrusTome transcriptome database, together with other published repositories, were used to identify *Met*, *Src*, *Kr-h1*, *E93*, *CBP* and *CtBP* transcripts across pancrustacean taxa, including some panarthropods (Table 1; Supplementary Data). Phylogenetic trees and MSAs were presented in the same format as that used for characterization of crustacean neuropeptide G protein-coupled receptors, insulin and growth factor receptors, and protein kinases A, C, and G [84,85,86,87]. The absence of some of these transcripts in certain species may be attributed to tissue source, RNA sequencing depth, and/or software and parameters used for transcriptome quality control, assembly, and filtering [79]. The sequences had the conserved domain organizations and motifs characteristic of each protein, which facilitated assignment to the correct gene family (Figure 2, Figure 3 and Figure 4, Figure 6, Figure 8, Figure 9, Figure 11, Figure 13, Figure 15, Figure 16 and Figure 17; Supplemental Data). The analysis established that insect Mblk-1 and crustacean E93 were orthologous HLH_Psq TFs (Figure 10). Contigs encoding each protein in each crustacean species, including isoforms that may have been generated by alternative splicing, appeared to be derived from one DNA sequence, which suggests that crustaceans have a single copy of *Met*, *Src*, *Kr-h1*, *E93*, *CBP* and *CtBP*. However, this must be confirmed by analysis of genomic sequences.

Met/Src is the MF receptor in crustaceans. In *Daphnia pulex*, Met/Src mediates transcriptional responses to MF [28]. Both Met and Src are members of the bHLH-PAS family of TFs, which have bHLH, PAS-A, PAS-B, and C-terminal transactivation domains (Figure 2 and Figure 7) [27]. The bHLH domain was highly conserved in decapods, with ten residues specifying DNA binding properties that were identical in all 19 species (Figure 3). MF initiates the assembly of the Met/Src receptor complex by binding to the Met PAS-B domain [28,97,98]. In *D. melanogaster*, eight conserved residues in the Met PAS-B domain mediate JH III binding, which results in dissociation of HSP83 and dimerization with Tai [27]. The Met/Tai heterodimer is stabilized by interactions between the bHLH/PAS-A/PAS-B regions of both proteins [27]. Interestingly, in the decapod and insect Met PAS-B domain, seven of the eight residues were identical (Figure 5) [69,70,72], which suggests that decapod and insect Met proteins differ in ligand binding affinities and specificities [17,28].

*Kr-h1* is a C_2_H_2_ Zf gene that is transcriptionally up-regulated by Met [36]. The domain organization is highly conserved in decapods (Figure 9). The number of C_2_H_2_ Zf repeats in the DNA-binding domain differed between insects with eight and crustaceans with seven (Figure 9 and Figure 10; [41,42,71]). Decapods and insects shared a KxFSVKNLxVHRR sequence in the second or third Zf repeat, respectively (Figure 10). The serine in that sequence is phosphorylated by PKCa in insects [13]. In the migratory locust (*Locusta migratoria*), methoprene increases phosphorylated Kr-h1 in juveniles and adults [13]. In larval and juvenile insects, phosphorylated Kr-h1 recruits CtBP to inhibit *E93* expression and metamorphosis [13]. In adults, phosphorylated Kr-h1 recruits CBP to stimulate ribosomal protein L36 expression and vitellogenesis [13]. These data suggest that the recruitment of CBP and CtBP by phosphorylated Kr-h1 is conserved in insects and decapods. Moreover, life history stage can determine the responses of tissues to MEKRE93 signaling. For example, in adult decapods, the MF-stimulated vitellogenesis by the hepatopancreas may be facilitated by recruitment of CBP by phosphorylated Kr-h1 to increase vitellogenin expression (reviewed in [7]; discussed below).

*E93* encodes a HTH_Psq transcription factor that is a downstream target of *Kr-h1* in insects [3,9,10,36,99,100]. A nuclear receptor interaction sequence (LxxLL or LLxxL) and CtBP interaction core motif (PxDLS) are located between the two HTH_Psq domains [78]. In decapod E93 proteins, these sequences are, respectively, LKHLL and PCDLS in *P. trituberculatus* [78], LKHLL and SCDLS in *C. maenas* (Supplemental Figures S10 and S22), and LRHLL and PCDLS in *G. lateralis* (Supplemental Figures S4 and S22).

MEKRE93 signaling is among several signaling pathways that regulate molting and YO ecdysteroid synthesis in decapods. Molt-inhibiting hormone (MIH) and other neuropeptides inhibit ecdysteroidogenesis and maintain the YO in the basal state during intermolt (reviewed in [1,7,101]. By contrast, MF can have the opposite effect as MIH. MF stimulates ecdysteroid production in the YO in vitro [74,78,102]. MF administration shortens the intermolt interval in intermolt animals by inducing the onset of premolt in multiple decapod species [76,103,104,105]. The ecdysteroid synthetic capacity of the YO is determined by the transcription and translation of Halloween genes *Neverland* (*Nvl*), *Spook* (*Spo*), *Phantom* (*Phm*), *Disembodied* (*Dib*), *Shadow* (*Sad*), and *Shed* (reviewed in [7]). In *G. lateralis*, *Gl-Sad* mRNA level is increased during mid- and late premolt when hemolymph 20E titers are elevated [106]. In *P. trituberculatis*, MF increases *Pt*-*Spo* and *Pt*-*Sad* expression in vivo and in vitro, which is mediated by *Pt*-*Met*, *Pt*-*Kr-h1*, and *Pt*-*E93* [78]. Activation of the YO in early premolt relies primarily on mTORC1-dependent translation, as molt induction has little or no effect on Halloween gene mRNA levels from intermolt to early premolt animals [106]. However, transition of the YO to the committed state in mid-premolt requires transcriptional control by mTORC1 and TGFβ/Activin signaling, as blocking with rapamycin or SB431542, respectively, inhibits increases in Halloween gene expression [106,107].

MEKRE93 signaling interacts with ecdysteroid-responsive genes to control Halloween gene expression in the YO. Binding of 20-hydroxyecdysone (20E) to the EcR/RXR ecdysteroid receptor initiates a cascade of nuclear receptor TFs; these are Broad Complex (Br-C), hormone receptor 3 (HR3), HR4, E74, E75, and Fushi tirazu factor 1 (Ftz-f1), which can be modulated by MF MEKRE93 signaling in target tissues [2,7,99,108,109,110,111]. Differential expression of EcR/RXR and ecdysteroid-responsive genes over the molt cycle suggest a feedback control of ecdysteroid synthesis when 20E titers are increasing during premolt [112,113]. In *P. trituberculatus* YO, RNAi knockdown of *Pt-E75* increased *Pt-Sad* expression, but had no effect on *Pt-EcR* and *Pt-RXR* expression [114]. E93 plays a central role as an inhibitor of ecdysteroid biosynthesis and gene expression, as *E93* RNAi knockdown increases *Pt-Spo* and *Pt-Sad* mRNA levels and ecdysteroid synthesis in the *P. trituberculatus* YO [78]. Moreover, MF and 20E have opposite effects on gene expression. MF inhibits *Pt-E93* expression and stimulates *Pt-Met*, *Pt-Kr-h1*, *Pt-Spo*, and *Pt-Sad* expression [78]. 20E stimulates *Pt-EcR* and *Pt-E93* expression and inhibits *Pt-Spo* and *Pt-Sad* expression (*Pt-Met* and *Pt-Kr-h1* mRNA levels were not determined) [78]. These data indicate that transcriptional control of YO ecdysteroidogenesis involves both 20E and MF signaling.

Ecdysteroid and MEKRE93 signaling also have roles in larval development and adult reproduction of decapods [2,7,67,115]. Both 20E and MF stimulate vitellogenesis in the hepatopancreas and ovarian growth and maturation in multiple decapod species [115,116]. Kr-h1 mediates MF stimulation of vitellogenin expression in the hepatopancreas [41,42,71]. As in insects, 20E and MEKRE93 signaling also control larval development and metamorphosis in decapods [9,117,118]. In *S. paramamosain*, increased expression of *Sp-Met* and *Sp-Kr-h1* occurs at the 5th-stage zoea to megalope and megalope to juvenile transitions [72]. MF blocks metamorphosis of 5th stage zoea and delays metamorphosis of megalope to juvenile stages [72,119]. RNAi knockdown of *Sp-Met* in 4th-stage zoea induces metamorphic phenotypes, which is correlated with reduced expression of *Sp-Met*, *Sp-Kr-h1*, *Sp-EcR*, and *Sp-E93* after 36 h [72].

Hormones and epigenetic mechanisms are tightly interconnected, with each shaping the other to fine-tune gene expression during insect development [120]. For instance, histone deacetylase 1 (HDAC1) plays a fundamental epigenetic role in *T. castaenum*, as JH reduces HDAC1 expression in a Met-dependent manner, thereby activating *Kr-h1* [121]. Several insect studies have reported that the MEKRE93 TFs undergo PTMs (e.g., phosphorylation, acetylation, and SUMOylation post-translational modifications), which in turn influence downstream effects. However, the specific target residues may vary among species. Phosphorylation impacts the interaction and transactivation of the Met receptor complex. In *A. aegypti*, JH triggers the phospholipase C (PLC) pathway, leading to the phosphorylation of Met, through the activation of calcium/calmodulin-dependent protein kinase II (CaMKII) that contains an auto-phosphorylation site at Thr286 [122]. Liu et al. [122] also identified 14 putative phosphorylation sites in Met and 23 phosphorylation sites in Tai. JH can also activate protein kinase C (PKC) in a PLC-dependent manner, which enhances DNA binding of the Met-Tai heterodimer to response elements [123]. Phosphorylation of specific residues in Met and Tai requires JH, which modulates Met/Tai function and increases binding to JH response elements (JHREs), whereas other residues are phosphorylated in the absence JH [91,122]. After JH exposure, *A. aegypti* Met residues Ser77 and Ser710 were phosphorylated, whereas phosphoserine residues at positions #73 and #747 were dephosphorylated [91]. In addition, JH induced a transient and reversible phosphorylation of Met at Thr664 and Ser723 [91]. In *H. armigera*, Met Thr393 phosphorylation is essential for Met-Tai heterodimerization, prevention of Met homodimerization, and binding to the E-box in the *Kr-h1* promoter [93]. Other putative phosphorylation sites were predicted in the Met PAS-B domain (Figure 5). In the red flour beetle (*Tribolium castaneum*), methoprene (a JH analog) exposure results in phosphorylation of Tai at S269 and of Met at T189, S191, S504, S438, S441, S459, T460, and T464 [124]. Potential phosphorylation sites in other regions of Met suggest that post-translational modifications modulate further regulation. Nuclear localization signals in the disordered C-terminus are straddled by phosphorylation sites, suggesting phosphorylation may regulate Met nuclear import and localization [124]. In *D. melanogaster*, Met phosphorylation promotes nuclear import mediated by heat shock protein 83 (Hsp83) and subsequent induction of *Kr-h1* transcription through *Kr-h1* promoter binding [125,126,127].

The E93/Mblk-1 orthologs have been reported to contain specific interaction sequences between the two HTH_Psq domains, where binding may occur to regulate gene transcription [128]. The identification of the nuclear receptor interaction sequence (LxxLL or LLxxL motif) in the E93/Mblk-1 sequences suggests that E93/Mblk-1 interacts with other ecdysteroid-responsive genes, such as E74, E75, and EcR [95]. The CtBP interaction sequence is conserved across pancrustaceans (except for *P. kraunhiae*), particularly the leucine residue associated with the activation of mTORC1, the signaling pathway driving ecdysteroidogenesis [49,107]. Phosphorylation of E93 is hypothesized to inhibit PI3K-mTORC1 signaling [49]. Crustacean E93/Mblk-1 sequences possess a potential PKC phosphorylation serine residue conserved across most insect orders (lepidopterans have a Ser–Thr substitution) [94]. Phosphorylation (or not) and localization of Mblk-1 in the honeybee brain is stage-specific [129]. In Hemiptera, the brown planthopper (*Nilaparvata lugens*) E93 retains the conserved serine in E93, whereas mealybugs exhibit reduced or delayed E93 expression in neotenic females, mediated by JH–dependent repression [130]. In neometabolous thrips (*Frankliniella occidentalis* and *Haplothrips brevitubus*), *E93* is upregulated during propupal and pupal stages and suppressed by JH analogs, demonstrating that shifts in the timing of *E93* expression under JH regulation contribute to the evolution of neometaboly [131]. Collectively, these data suggest that post-translational modifications of E93/Mblk-1 modulate interactions with other TFs and transcriptional co-regulators that are stage-specific in both crustaceans and insects.

By regulating the expression of TFs, CBP serves as a central modulator of insect JH and ecdysteroid signaling pathways. In *A. aegypti*, RNAi-mediated knockdown of CBP downregulated *Kr-h1* and upregulated *E93* in the JH and ecdysteroid pathways, respectively, which resulted in premature metamorphosis, larval-pupal intermediate formation, improper eye development, and increased mortality [132]. JH increased acetylation of core histones at the *Kr-h1* promoter region and increased *Aa-Kr-h1* expression, while CBP inhibited *E75a*-dependent expression of other ecdysteroid-responsive genes (e.g., *Aa-EcR*, *Aa-USP*, *Aa-Br-C,* and *Aa-E93*) [132]. In *T. castaneum*, *Tc-Kr-h1* expression decreased when CBP was knocked down, but was increased with the presence of a HDAC inhibitor [133]. Roy et al. [134] showed that RNAi-mediated knockdown of CBP in *T. castaneum* decreased the expression of JH responsive genes (e.g., *Kr-h1* and *Hairy*) and ecdysteroid-responsive genes (e.g., *EcR*, *E74*, *E75*, and *Br-C*). In CBP RNAi experiments, molting was delayed in pre-metamorphic German cockroaches (*B. germanica*) via reduced food intake and downregulating ecdysteroid signaling genes *E75a/b* and *HR3* [135]. Although CBP could affect molting directly via ecdysteroid-responsive genes, it is plausible that the delay resulted from CBP acting indirectly through TGFβ and/or JH signaling. Fernandez-Nicolas and Belles [135] reported that the downregulation of CBP disrupted the normal *Kr-h1* decline and increase in *E93*. Post-dsCBP treatment with JH abrogated the normal JH-induced increase in *Kr-h1* expression and reduced *E93* expression. This suggests that CBP is a co-activator to *Kr-h1*, *E93*, or both [135]. In *B. germanica*, PKCα phosphorylates Kr-h1 and recruits CBP to enhance expression of genes associated in vitellogenesis in adults, but recruits CtBP in juveniles to repress *E93* expression [13]. Some post-translational modifications may be conserved in crustaceans, potentially affecting downstream TF functional interactions.

## 4. Materials and Methods

Previously identified and/or annotated protein sequences of the MEKRE93 transcription factor network and transcriptional co-regulators were used as queries in the National Center for Biotechnology Information Basic Local Alignment Search Tool (NCBI BLAST) program to search for candidate genes in CrusTome, CrustyBase, CrusTF (Crustacean Transcription Factor), CAT (Crustacean Annotated Transcriptome), and GenBank databases [79,80,81,82,83,136]. The following sequences were used for the initial search queries: *E. sinensis* Met (GenBank accession QLH01997.1), *T. castaneum* Src (BAN62669.1), *P. trituberculatus* Kr-h1, *L. vannamei* E93/Mblk-1 (accession IOCAS.LVAN11361 from [136], *B. germanica* CBP (CUT08824.1), and *L. vannamei* CtBP (AOD27417.1). Reciprocal BLAST searches were conducted to verify sequence identities. Reference sequences and the BLAST hit queries were aligned with Multiple Alignment using Fast Fourier Transform (MAFFT; version 7.490) employing long, iterative global refinement with global pairwise strategy (L-INS-I; --genafpair --maxiterate 10,000) with the DASH parameter (--dash) to incorporate structural homolog information [137,138]. Only the original sequences were retained in the final multiple sequence alignment (MSA; --originalseqonly); the alignment was performed using six threads (--thread 6). The MSAs were trimmed with ClipKIT using the *smart-gap* parameter to remove gaps, while preserving phylogenetically informative sites [139]. Phylogenetic analyses were inferred from the aligned protein sequences with the suitable model, as determined by ModelFinder following the Bayesian Information Criterion (BIC) [140,141] (https://tree.bio.ed.ac.uk/software/figtree/). Maximum likelihood phylogenetic trees were constructed using IQ-Tree 2 using 1000 ultrafast bootstrap iterations (UFBoot = 1000) [142,143]. The phylogenetic trees were then rooted and visualized in FigTree (version 1.4.4) and Inkscape (https://gitlab.com/inkscape/inkscape).

Conserved domains, motifs, and residues were identified with the NCBI ConservedDomain Database (CDD) platform (https://www.ncbi.nlm.nih.gov/Structure/cdd/wrpsb.cgi). For further contig annotation, MSAs were executed with Mafft with local pairwise-alignment with consistency scoring and iterative refinement (E-INS-I) and default vacancy penalty scores and parameters on the Jalview (version 2.11) platform and subsequently trimmed with ClipKIT [138,139,144,145,146]. Domain organizations were illustrated and annotated using Illustrator for Biological Sequences 2.0 [147] (https://ibs.renlab.org/#/home). NetPhos 3.1 web server (https://services.healthtech.dtu.dk/services/NetPhos-3.1/) was used to identify putative serine, threonine, and tyrosine phosphorylation sites [148,149]. Species taxonomic ranks were annotated according to the classifications defined by Giribet and Edgecombe [150].

## 5. Conclusions

This study constitutes the most comprehensive catalog of crustacean MF MEKRE93 signaling and co-regulator genes to date. Phylogenetic and sequence analysis revealed high conservation of the domain organizations and functional motifs, which aided in the identification and annotation of new sequences from transcriptomes and genomes in GenBank and other repositories. Transcriptome databases, such as CrusTome, CrustyBase, CrustTF, and CAT, have demonstrated to be useful resources for examining gene evolution and conservation of gene function, as well as providing structural information crucial to the study and analysis of physiological processes in Crustacea [7].

MF and ecdysteroid signaling pathways control YO ecdysteroidogenesis. Under certain conditions, such as in intermolt animals, MF can stimulate YO ecdysteroid secretion [74,78] and MF injection can accelerate molting [75,76], as reviewed in [7]. Moreover, the YO expresses MF biosynthetic enzymes, suggesting that MF acts as an autocrine factor [107]. Ecdysteroid receptor (*EcR*/*RXR*) and ecdysteroid-responsive genes *Br-C*, *E74*, *E75*, *HR3*, *HR4*, and *Ftz-f1* are expressed in the decapod YO, suggesting a 20E feedback on transcriptional control of Halloween genes, such as *Spo* and *Sad* [78,106,112,114,151,152]. During premolt, mTORC1 and TGFβ signaling pathways are required for increased ecdysteroid production in the YO (reviewed in [7,101]). This is analogous to insects, in which mTORC1, TGFβ, JH, and 20E signaling pathways control ecdysteroid synthesis in the prothoracic gland [36,153,154,155,156].

## Figures and Tables

**Table 1:**
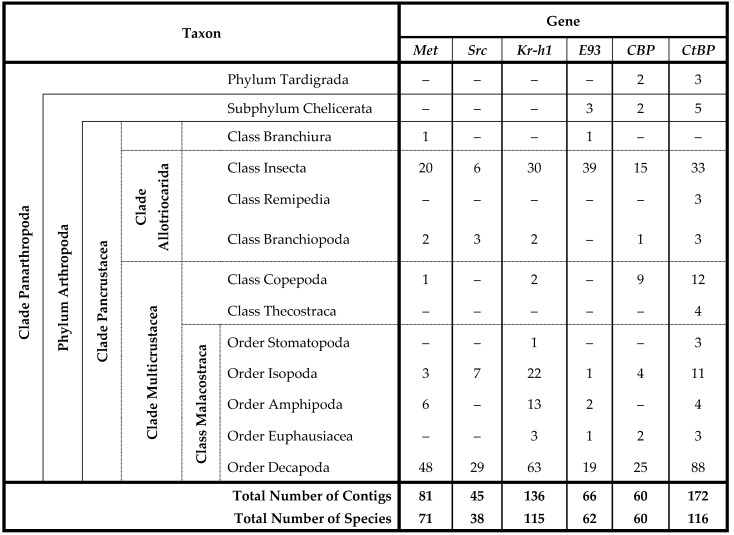
Taxonomic distribution of MEKRE93 transcription factor and transcriptional co-regulators CBP and CtBP sequences. Summary of the number of sequences obtained across Clade Panarthropoda. Abbreviations: CBP, CREB-binding protein; CtBP, C-terminal-binding protein; E93, Ecdysone response gene 93; Kr-h1, Krüppel homolog 1; Met, Methoprene-tolerant; and Src, Steroid receptor coactivator.

**Table 2:** MEKRE93 signaling and co-regulator transcripts from the *G. lateralis* Y-organ (Y) transcriptome [79]. Abbreviations: aa, amino acids; bp, base pairs; bHLH-PAS, basic helix-loop-helix–Per-Arnt-Sim; C_2_H_2_ Znf, Cys_2_-His_2_ zinc finger; HTH_Psq, helix-turn-helix, pipsqueak; ORF, open reading frame; and UTR, untranslated region. Asterisks (*) indicate partial sequences (incomplete ORF). Minus (−) indicates that the UTR is missing in the partial contig sequence.

Gene	Class	Contig #	Length (bp)	ORF (aa)	UTR (bp)	GenBank Accession
*Gl-Met*	bHLH-PAS	Y EVm001315t1	3511	1001	**5′:** 177 **3′:** 327	PQ306373
*Gl-Src*	bHLH-PAS	Y EVm002648t2	3179	707 *	**5′:** 1056 **3′:** −	PQ308086
*Gl-Kr-h1*	C_2_H_2_ Znf	Y EVm003411t1	2476	616	**5′:** 345 **3′:** 279	PQ306361
*Gl-E93*	HTH_Psq	Y EVm001885t1	3114	854 *	**5′:** − **3′:** 548	PQ306469
*Gl-CBP*	Transcriptional co-activator	Y EVm000165t1	7188	2197	**5′:** 54 **3′:** 540	PQ306374
*Gl-CtBP*	Transcriptional co-repressor	Y EVm005788t1	1905	454	**5′:** 427 **3′:** 113	PQ306376

**Table 3:** MEKRE93 signaling and co-regulator transcripts from the *C. maenas* Y-organ (Y) and central nervous system (CNS) transcriptomes [79]. Abbreviations: aa, amino acids; bp, base pairs; bHLH-PAS, basic helix-loop-helix–Per-Arnt-Sim; C_2_H_2_ Znf, Cys_2_-His_2_ zinc finger; HTH-Psq, helix-turn-helix, pipsqueak; ORF, open reading frame; and UTR, untranslated region. Asterisks (*) indicate partial sequences (incomplete ORF). Minus (–) indicates that the UTR is missing in the partial contig sequence.

Gene	Class	Contig #	Length (bp)	ORF (aa)	UTR (bp)	GenBank Accession
*Cm-Met*	bHLH-PAS	Y EVm001443t1	3677	998 *	**5′:** 682 **3′:** −	PQ306465
		CNS EVm002019t1	2986	921 *	**5′:** 223 **3′:** −	PQ321205
*Cm-Src*	bHLH-PAS	Y EVm000958t2	4878	1185 *	**5′:** 1323 **3′:** −	PQ327774
		CNS EVm001140t3	5299	1174 *	**5′:** 1777 **3′:** −	PQ327775
*Cm-Kr-h1*	C_2_H_2_ Znf	Y EVm004068t1	3842	603	**5′:** 1425 **3′:** 603	PQ306239
		CNS EVm004576t1	3418	603	**5′:** 1020 **3′:** 586	PQ317097
*Cm-E93*	HTH_Psq	Y EVm001380t1	4185	1016	**5′:** 158 **3′:** 975	PQ306467
		CNS EVm001710t1	3880	989	**5′:** 35 **3′:** 875	PQ317110
*Cm-CBP*	Transcriptional co-activator	Y EVm000144t1	7455	2321 *	**5′:** 490 **3′:** −	PQ306466
		CNS EVm000162t1	7435	2321 *	**5′:** 470 **3′:** −	PQ327773
*Cm-CtBP*	Transcriptional co-repressor	Y EVm011142t2	1100	278 *	**5′:** − **3′:** 198	PQ327771
		CNS EVm011952t2	1008	326 *	**5′:** 28 **3′:** −	PQ327772

**Table 4:** Consensus sequence motifs in the C-terminal domain (CTD) of the C-terminal-binding protein (CtBP) transcriptional co-repressor. Aromatic residues, or likely animo acid substitutions, are bolded in red.

Taxon	Motif Consensus Sequences			
	Motif #1	Motif #2	Motif #3	Motif #4
Brachyura	CVNKEY	G**V**NG–YY	VHSTT	
Anomura	CVNKEY	G**V**NG–YY or G**V**NG–Y**F**	VHSTT	
Achelata	CVNKEY	G**V**NG–YY	VHSTT	
Astacidea	CVNKEY	G**V**NG–YY	VHSTT	
Dendrobranchiata	CVNKEY	G**V**NG–YY	VHSTT	
Caridea	CVNKEY	G**V**NG–YY or **SV**NG–YY	VHSTT	
Amphipoda	CVNKEY	G**V**NG–Y**F**	VHSTT/VHST**A**	
Isopoda	CVNKEY	G**V**NG–YY	VHSTT	
Copepoda	CVNKEY or C**I**NKEY	G**M**NG–YY or GLNG–YY/G**P**N**S**–YY	**A**HSTT	
Euphausiacea	CVNKEY	G**V**NG–YY	VHSTT	
Cladocera	CVNKEY	GLNG–YY	**A**HS**SA**	
Stomatopoda	CVNKEY	G**V**NG–YY	VHSTT	
Notostraca	CVNKEY	GLNG–YY	**A**H**NSA**	
Thecostraca	CVNKEY	GLNG–YY	**A**HST**S**	S**DI**H

## Data Availability

The original contributions presented in this study are included in the article/Supplementary Material. Further inquiries can be directed to the corresponding authors.

## Supplementary Materials

The following supporting information can accessed on the Harvard Dataverse online repository (Bentley, Vanessa, 2025, “Methyl farnesoate”, https://doi.org/10.7910/DVN/BCG5V4, Harvard Dataverse, V1.
